# Systematic analysis of hematopoietic gene expression profiles for prognostic prediction in acute myeloid leukemia

**DOI:** 10.1038/srep16987

**Published:** 2015-11-24

**Authors:** Frederick S. Varn, Erik H. Andrews, Chao Cheng

**Affiliations:** 1 Department of Genetics, Geisel School of Medicine at Dartmouth, 1 Rope Ferry Road, Hanover, New Hampshire 03755, USA; 2 Institute for Quantitative Biomedical Sciences, Geisel School of Medicine at Dartmouth, One Medical Center Drive, Lebanon, New Hampshire 03766, USA; 3 Norris Cotton Cancer Center, Geisel School of Medicine at Dartmouth, One Medical Center Drive Lebanon, New Hampshire 03766, USA

## Abstract

Acute myeloid leukemia (AML) is a hematopoietic disorder initiated by the leukemogenic transformation of myeloid cells into leukemia stem cells (LSCs). Preexisting gene expression programs in LSCs can be used to assess their transcriptional similarity to hematopoietic cell types. While this relationship has previously been examined on a small scale, an analysis that systematically investigates this relationship throughout the hematopoietic hierarchy has yet to be implemented. We developed an integrative approach to assess the similarity between AML patient tumor profiles and a collection of 232 murine hematopoietic gene expression profiles compiled by the Immunological Genome Project. The resulting lineage similarity scores (LSS) were correlated with patient survival to assess the relationship between hematopoietic similarity and patient prognosis. This analysis demonstrated that patient tumor similarity to immature hematopoietic cell types correlated with poor survival. As a proof of concept, we highlighted one cell type identified by our analysis, the short-term reconstituting stem cell, whose LSSs were significantly correlated with patient prognosis across multiple datasets, and showed distinct patterns in patients stratified by traditional clinical variables. Finally, we validated our use of murine profiles by demonstrating similar results when applying our method to human profiles.

Hematopoiesis is the developmental program that gives rise to the cellular components of blood. This process is hierarchical, with multipotent hematopoietic stem cells (HSCs) generating myeloid and lymphoid progenitor cells, which then generate more differentiated cell types^1^. Mutations in hematopoietic cells can upset this process, resulting in a variety of blood maladies, including cancer. Acute myeloid leukemia (AML) is one such cancer, characterized by the accumulation of aberrant primitive myeloid cells^2^,^3^. These cells have a limited proliferative capability, suggesting the existence of an underlying sub-population of proliferative cells that maintain the leukemia. Evidence for the existence of these leukemia stem cells (LSCs) was first reported when a population of human CD34^+^CD38^−^ AML patient cells successfully initiated AML in a SCID mouse xenograft model^4^,^5^,^6^. A follow-up study showed that LSCs are hierarchically organized, with varying self-renewal capabilities^7^. Additional reports have suggested that AML-initiating cells are not limited to the CD34^+^CD38^−^ cell population and are found in the CD34^+^CD38^+^ and CD34^−^CD38^−^ cell populations as well^8^,^9^. Taken together, these studies portray AML as a heterogeneous disease that can be initiated and maintained through a variety of cell types.

The heterogeneity of AML has made accurately predicting patient prognosis difficult. Most commonly, cytogenetic analysis is used to subtype patients by karyotype^10^. However, nearly 50% of AML cases have a normal karyotype, leaving a large fraction of heterogeneous samples without further classification. For these patients, molecular mutations, such as *FLT3* internal tandem duplications, *MLL* partial tandem duplications, mutations in the *NPM1* and *CEBPA* genes, and heightened expression of the *BAALC* and *ERG* genes are used as prognostic indicators^11^. Gene expression microarray analyses have led to additional classification schemes that can further stratify patients for prognostic purposes and increase the resolution by which we can study their molecular pathology. Multiple studies have clustered AML samples using gene expression profiles, revealing different molecular subgroups defined by previously identified cytogenetic abnormalities^12^,^13^,^14^,^15^,^16^,^17^,^18^. Another study has drawn upon the stem cell-like nature of LSCs to create a robust, prognostic gene expression signature^19^. Patients with high expression of this signature’s genes tend to have an adverse outcome, supporting the LSC hypothesis and suggesting that LSC activity may be driving AML severity. This idea was furthered by work showing that HSCs and LSCs share a core transcriptional program that imparts stem cell properties, including self-renewal and differentiated progeny production^20^.

Although these studies have improved prognostic accuracy, they have limitations. Hematopoiesis is extremely complex, involving a vast number of cells of varying maturity that express a wide array of cell-surface markers^1^. Understanding the dominant hematopoietic programs in a patient’s leukemia may elucidate features that can be used to improve disease characterization. Recently, a group has reported that, in *MLL*-rearranged AML, the LSC shares a common transcriptional signature similar with its cell of origin. This group compared AML arising from HSCs to AML arising from granulocyte-macrophage progenitors (GMPs) and found that HSC-derived AML had an expedited onset and a greater resistance to chemotherapy^21^. Taking this idea further, a systematic analysis that correlates AML tumor profiles across profiles from cells throughout the hematopoietic hierarchy could offer a highly specific assessment of the molecular makeup of a patient’s leukemia, including any hematopoietic programs the cell may have inherited from it’s origin cell. While human hematopoietic gene expression profiles are a useful tool to use in this analysis, murine hematopoietic profiles can serve as a proxy for many human profiles^22^,^23^ and offer several additional advantages. Using mice allows for hematopoietic gene expression profiling to be carefully controlled to diminish noise and batch variability between studies. Additionally, mice can be easily subjected to genetic and environmental perturbations enabling more comprehensive profiling studies of hematopoiesis. One such study has been performed by the Immunological Genome Project Consortium and has resulted in a series of 232 murine hematopoietic cell lineage gene expression profiles^24^.

Here, we extend our previously developed BASE (binding association with sorted expression) algorithm^25^ to systematically investigate the similarity between human AML gene expression data and the 232 murine transcription profiles compiled by the Immunological Genome Project. The BASE algorithm was originally developed to infer transcription factor activity from gene expression profiles using ChIP-chip and ChIP-seq data. We have recently shown that it can be used to calculate the activity of binary gene sets in breast cancer samples^26^ and now, by substituting binding affinity data with hematopoietic gene expression profiles, we demonstrate its utility in calculating the activity of hematopoietic transcription programs in patient AML samples. This process will provide information about the molecular makeup of a patient’s AML, allowing us to perform follow-up analyses to make prognostic predictions and further characterize the cancer.

We begin by using BASE to calculate the lineage similarity score (LSS), a summary statistic that encapsulates the similarity between patient AML gene expression data and the murine hematopoietic cell lineage expression profiles generated by the Immunological Genome Project. We then apply Cox proportional-hazards (PH) models to identify the hematopoietic cell types whose LSSs most closely associate with patient survival. We find that patients whose AML profiles most resemble immature hematopoietic cell profiles tend to have diminished survival time. As a proof of concept, we highlight a short-term reconstituting stem cell gene expression profile that is especially predictive of patient prognosis. We demonstrate that this profile aligns with traditional AML classification schemes including French-American-British (FAB) subtyping and *FLT3* mutation status, and is also predictive of induction therapy response. We apply this profile to three independent AML patient datasets and show that it remains predictive across all of them. Finally, we validate our findings using murine profiles by comparing their prognostic performance with that of their analogous human cell counterparts.

## Results

### Overview

**Figure 1:**
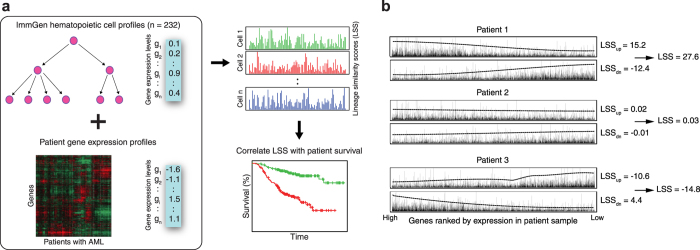
Overview of workflow. (**a**) Murine hematopoietic cell expression profiles were downloaded from the Immunological Genome Project and compared against patient gene expression profiles from an AML dataset of interest using the BASE algorithm. This resulted in a lineage similarity score (LSS) that reflects gene expression concordance between a given hematopoietic cell type and a given patient. The resulting patient LSSs were then used as covariates in a Cox proportional hazards model. Cell types that were significantly associated with patient survival were explored in more detail. (**b**) For each murine hematopoietic cell profile, genes are ranked from high to low based on their expression values. These weights are then assigned to a list of genes ranked by patient gene expression profiles. LSS_up_ is determined based on concordance between hematopoietic up-regulated weights and patient rank, with a more positive value representing higher concordance. LSS_dn_ is determined based on concordance with the down-regulated weights and patient rank, with a more negative value representing higher concordance. Dotted lines represent 10*mean(weight) over a rolling window of 1000 genes. The LSS_dn_ is then subtracted from the LSS_up_ to obtain the final LSS, which represents the similarity between patient and hematopoietic cell gene expression profiles. Patients 1, 2, and 3 are examples of a high, intermediate, and low LSS, respectively.

Figure 1A provides a schematic of our analysis. Murine hematopoietic cell lineage profiles for 232 cell types from the Immunological Genome Project^24^ were combined with the AML patient gene expression dataset generated by Bullinger *et al.* (GSE 425)^27^ using BASE to generate a matrix of LSSs for each patient across all 232 cell types. A high LSS indicated high concordance between a patient’s and a cell type’s gene expression profile, while a low LSS indicated the opposite (Fig. 1B). Univariate survival analyses using Cox PH regression were conducted for each cell type’s LSSs to identify cell types with AML prognostic significance. Multivariate Cox PH survival analyses to correct for clinicopathological variables were then conducted on these cell types for further analysis.

### Identification of survival-associated hematopoietic cell profiles

**Figure 2:**
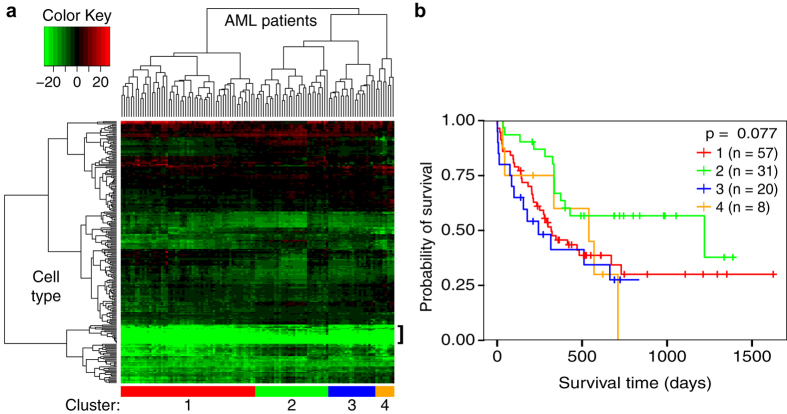
Exploratory analysis of Immunological Genome Project LSSs. (**a**) Heatmap showing the pattern of 232 LSSs across the 116 samples from the Bullinger dataset. Each column represents one patient’s LSS profile for each of the 232 murine hematopoietic cell types from the Immunological Genome Project. Green is indicative of a lower (less similar) LSS while red is indicative of a higher (more similar) LSS. Patients tended to have lower LSSs in more differentiated cell types (bracket). Patient clusters were chosen based on patient location in the heatmap dendrogram (sidebar). (**b**) Kaplan-Meier plot depicting the survival probability over time for each cluster. Vertical hash marks indicate points of censored data. The four clusters did not show a significant difference in survival time (p > 0.05).

To identify patterns of hematopoietic cell types that correlate with overall AML patient survival, hierarchical clustering and heatmap generation using the patient LSSs for each cell type was performed (Fig. 2A). Most of the 232 murine cell types did not show any concordance in gene expression with AML patients. Notably, the lowest LSSs were found in a cluster enriched in differentiated myeloid and stromal cell types, indicating high discordance between AML patient profiles and cells of these types (Fig. 2A, bracket). However, there were 42 cell types that had an LSS > 0 in at least half of the samples, showing that many murine hematopoietic lineages do share biological properties with human leukemias, despite the species barrier.

To examine whether this similarity had a clinical application in AML, we cut the hierarchical clustering tree at a depth that generated four clusters of samples with low intra-cluster LSS variability but high inter-cluster LSS variability. We then compared the survival distributions of each cluster using a log-rank test (*P* = 0.08) (Fig. 2B). While the survival distributions of each cluster were not significantly different from each other, we decided to investigate further by looking at the survival distributions of the individual cell type LSSs.

**Figure 3:**
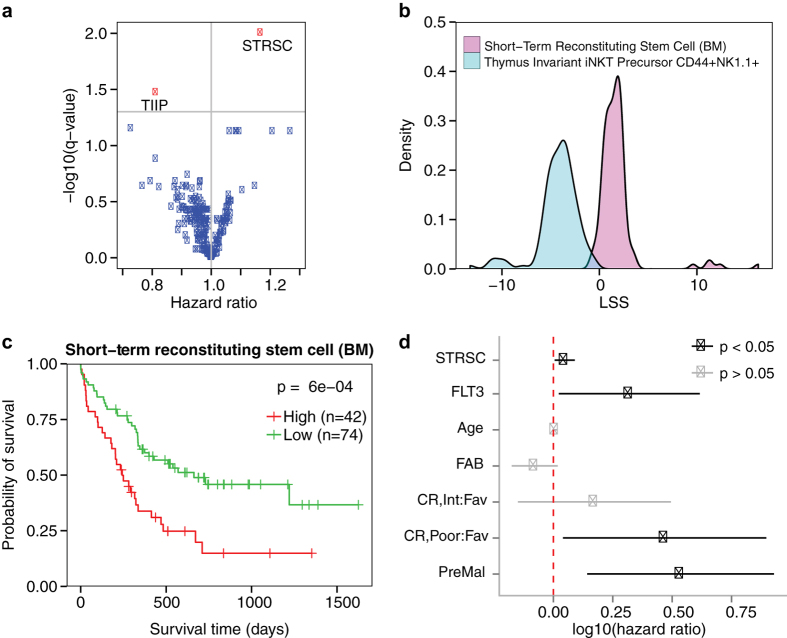
Survival analysis of the short-term reconstituting stem cell LSS. (**a**) Distribution of the hazard ratios and adjusted p-values derived from univariate Cox proportional-hazards models that included murine hematopoietic cell type LSSs as the variables. Each point corresponds to a different cell type, with red points corresponding to cell types with an adjusted p-value < 0.05 and blue points corresponding to cell types with an adjusted p-value > 0.05. The two red dots correspond to the short-term reconstituting stem cell (STRSC) and thymus invariant iNKT precursor CD44^+^NK1.1^+^ (TIIP). (**b**) Density plot of the LSSs for the two cell types significantly associated with survival in the Bullinger dataset. The STRSC is represented by a cyan curve and the TIIP is represented by a magenta curve. (**c**) Kaplan-Meier plot depicting the survival probability over time for samples with a high (red curve) and low (green curve) STRSC LSS. Vertical hash marks indicate points of censored data. (**d**) In a multivariate Cox proportional-hazards model, the STRSC LSS is significantly predictive of patient survival even after adjusting for traditional clinical factors. Bars represent log(hazard ratio) 95% confidence interval. Red dotted line indicates where the log10(hazard ratio) = 0.

Univariate Cox PH models were used to measure the correlation between LSS as a continuous variable and patient survival time for each cell type (Supplementary Table S1). Figure 3A shows the distribution of the adjusted *P* and hazard ratios (HR) of each cell type’s result. Only two cell types yielded adjusted *P* < 0.05, the CD150^−^CD48^−^ short-term reconstituting stem cell (STRSC) derived from bone marrow (adjusted *P* = 0.01, HR = 1.17, 95% CI = 1.08–1.25) and the CD44^+^NK1.1^+^ thymus invariant iNKT precursor cell (adjusted *P* = 0.03, HR = 0.81, 95% CI = 0.72–0.91). Figure 3B shows their LSS distributions. The STRSC LSSs were primarily positive, with 111/116 samples having a positive LSS, while all 116 samples had negative CD44^+^NK1.1^+^ thymus invariant iNKT precursor cell LSSs. Because we were primarily interested in the survival distribution of leukemias that share expression profiles with cell types, we decided to examine the STRSC profile in more detail.

Kaplan-Meier estimators were fitted to two groups of samples stratified into high and low LSS groups (Fig. 3C). A log-rank test revealed that the patients in the high group had significantly shorter survival times than the patients in the low group (*P* = 6e-4). This result was robust to a multivariate Cox PH model correction for age, FAB subtype, *FLT3* mutation status, CALGB-defined cytogenetic risk category, and history of preceding malignancy (*P* = 0.03, HR = 1.12, 95% CI = 1.01–1.23) (Supplementary Table S2; Fig. 3D). Taken together, this suggested that patients whose leukemias’ genetic profiles more closely reflected the genetic profile of the STRSC tended to die at a faster rate than patients with more dissimilar profiles.

### Cell lineage similarity scores and survival time

**Figure 4:**
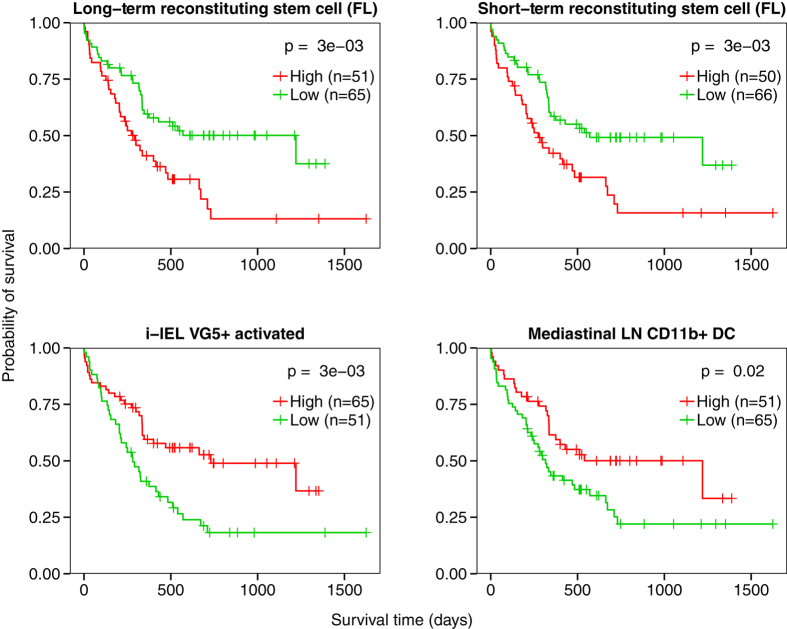
Survival analysis of different LSSs in AML patients. Kaplan-Meier plots depicting the survival probability over time for samples with high (red curves) and low (green curves) LSSs for four different cell types. Vertical hash marks indicate points of censored data. Hazard ratios are > 1 in the long-term reconstituting stem cell from fetal liver and short-term reconstituting stem cell from fetal liver cell types, indicating genetic similarity to these cell types has a deleterious effect on survival (top). Hazard ratios are < 1 in i-IEL VG5^+^, activated and lung CD11b^+^ DC, indicating greater profile similarity to these cell types is associated with increased survival time (bottom).

To look at whether this effect was localized to the STRSC profile, we relaxed the cutoff of our analysis and examined all lineages that were correlated with patient survival at an unadjusted *P* < 0.05 threshold. To identify cell types that shared similar characteristics with the STRSC profile, we separated the significant cell types into a HR > 1 and a HR < 1 group. Strikingly, we found that 9/11 cell types in the HR > 1 group were classified as stem cells by the Immunological Genome Project. The two remaining lineages were both located early in the T cell development pathway^28^. We highlight two examples in this group, the CD150^+^CD48^−^ long-term reconstituting stem cell (LTRSC) derived from fetal liver and the CD150^−^CD48^−^ STRSC derived from fetal liver (Fig. 4, top). When samples were stratified into high and low LSS groups for each lineage, the samples with higher LSSs had significantly worse survival than those with lower LSSs (*P* = 3e-3 and 3e-3, respectively, log-rank test).

In contrast, gene expression profiles that yielded a HR < 1 tended to be from more differentiated cell types. This group included many different cell types, including dendritic cells (DC), monocytes, natural killer cells, active and inactive αβ T cells, and γδ T cells. Notably, none of the cells in this group were stem cells. When samples were stratified by LSS for two examples from this group, the Vg5+ intestinal intraepithelial lymphocyte (i-IEL) and the lung CD11b^+^ DC, the samples with higher LSSs had significantly better survival than those with lower LSSs (*P* = 3e-3 and *P* = 0.02, respectively, log-rank test) (Fig. 4, bottom). As a negative control to these analyses, we examined the association between patient survival and stromal cell LSS, as these scores were uniformly distributed across samples (Fig. 2A, bracket). As expected, none of these cell types were significantly associated with patient survival (*P* > 0.1, Cox PH, Supplementary Table S1).

### Association of STRSC LSSs with clinical variables

**Figure 5:**
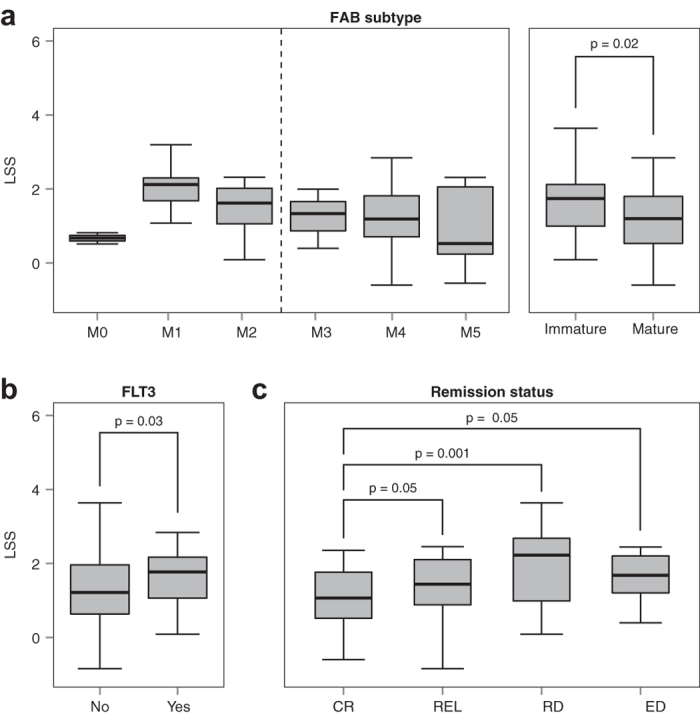
Association of STRSC LSS with traditional clinical variables. (**a**) STRSC LSS distributions across the different FAB subtypes (M0-M5) available in the Bullinger dataset. There was an n > 12 for each subtype, except for M0, where n = 3. When FAB subtype was dichotomized into immature (M0-M2) and mature (M3-M5) subgroups, there was a significant difference in the LSS between the two groups (*P* = 0.02, Wilcoxon rank-sum test). (**b**) LSS distributions across samples without and with a *FLT3* mutation. LSSs were significantly higher in the yes group than the no group (*P* = 0.03, Wilcoxon rank-sum test). (**c**) LSS distributions for patients that had different chemotherapy outcomes. Patients with complete response (CR) had significantly lower LSSs than patients with relapse (REL), refractory disease (RD) and early death (ED) (*P* = 0.05, 0.001, and 0.05, respectively, Wilcoxon rank-sum test). For all panels, box spans quartiles, with line representing median. Outliers were not removed. Whiskers represent absolute range excluding outliers.

The FAB subtype system uses the percentage and morphology of myeloblasts and erythroblasts in a patient’s blood to subtype the patient’s disease. The system classifies AML into one of eight subtypes, M0 through M7. The M0 through M5 subtypes have high percentages of immature myeloblasts, with M0 myeloblasts appearing the least mature and M5 myeloblasts appearing the most mature histologically, while the M6 and M7 subtypes have high percentages of immature erythrocytes and megakaryocytes, respectively^29^. The leukemia samples in this dataset included 3 M0, 13 M1, 25 M2, 12 M3, 35 M4, and 15 M5. We examined the STRSC LSS distributions of the M0-M5 subtypes (Fig. 5A). As can be seen, the LSSs demonstrated a decreasing trend from M1 to M5, suggesting that that the mouse STRSC LSS reflected the degree of differentiation in human AML. In support of this, a significant difference in LSS was observed between the subtypes (*P* = 0.02, Kruskal-Wallis). Furthermore, dichotomizing patient samples into two groups on the basis of cellular maturity, with the immature group made up of the M0 through M2 subtypes and the mature group consisting of the M3 through M5 subtypes, revealed that samples from more immature FAB subtypes had significantly higher STRSC LSSs than samples from more mature FAB subtypes (*P* = 0.02, Wilcoxon rank-sum test) (Fig. 5A). Though the M0 subtype was expected to have the highest STRSC LSS, the low number of M0 samples may have been confounding this observation. Interestingly, we observed a decreasing trend from M0 to M5 for LSSs from naïve CD8+ and CD4+ T cells that reside in the lymph node (Supplementary Fig. S1). However, LSSs of these cell types were not associated with patient survival.

*FLT3* is a receptor tyrosine kinase involved in the proliferation and development of hematopoietic stem cells^30^. Mutations resulting in *FLT3* activation have been found in about 30% of AML patients and are correlated with poor prognosis^30^. Interestingly, samples with a mutated copy of *FLT3* had significantly higher STRSC LSSs than *FLT3* wild type samples (*P* = 0.03, Wilcoxon rank-sum test, Fig. 5B). This result indicated that there was a correlation between the mouse STRSC LSS and the mutation status of the *FLT3* gene.

Stratifying patients by remission status allowed us to test whether there was a correlation between STRSC LSSs and the patient response to induction therapy^27^. There were four types of responses observed: complete response to therapy (CR), relapse (REL), refractory disease (RD), and early death due to treatment toxicity (ED). A significant difference in LSS was observed across the treatment outcomes (*P* = 0.005, Kruskal-Wallis). Additionally, STRSC LSSs in CR samples were significantly lower than REL, RD, and ED samples (*P* = 0.05, 0.001, and 0.05, respectively, Wilcoxon rank-sum test; Fig. 5C). These results suggested that the STRSC LSS could be a useful marker to predict the effectiveness of induction therapy on a patient.

### Application of the STRSC to other datasets

**Figure 6:**
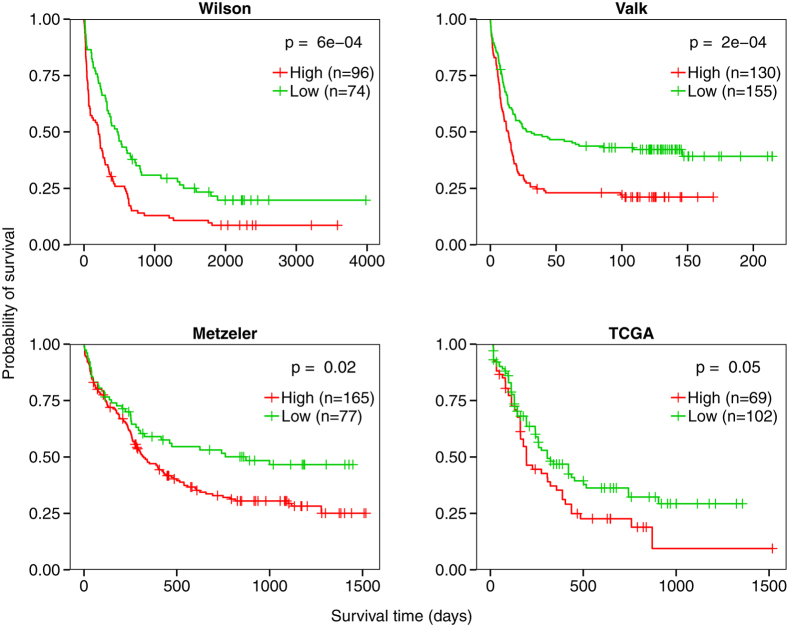
Survival analysis of the STRSC LSS across datasets. Across all datasets tested, patients with high STRSC LSSs (red curve) had significantly shorter survival times than those with low STRSC LSSs (green curve) (all *P* < 0.05, log-rank test). Vertical hash marks indicate points of censored data.

To confirm the findings from the Bullinger dataset, we expanded our analysis to four additional independent datasets by Wilson *et al.* (willm-00119)^31^, Valk *et al.* (GSE1159)^15^, Metzeler *et al.* (GSE12417)^32^, and the AML dataset generated by The Cancer Genome Atlas (TCGA)^33^. For each dataset, patients were stratified into high and low groups based on their STRSC LSS. This profile remained predictive of overall survival across the Wilson (*P* = 6e-5, log-rank test), Valk (*P* = 2e-5, log-rank test), Metzeler (*P* = 0.02, log-rank test), and TCGA (*P* = 0.05, log-rank test) datasets (Fig. 6). The reproducibility of the STRSC profile validated our findings in the Bullinger dataset and indicated that the results from our analysis were generalizable across multiple datasets.

### Comparison of murine hematopoietic profiles to human hematopoietic profiles

**Figure 7:**
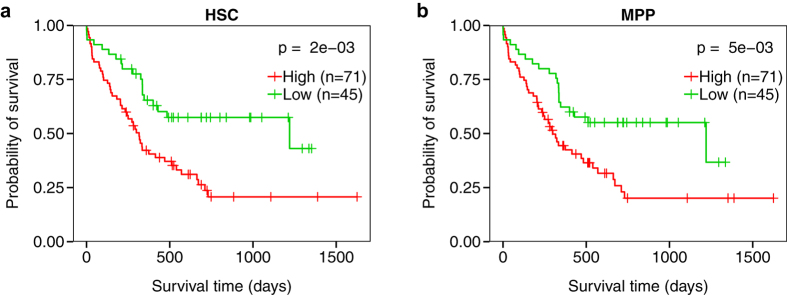
Survival analysis of LSSs for human hematopoietic cell types. Kaplan-Meier plots depicting the survival probability over time for samples with high (red curves) and low (green curves) LSSs for (**a**) human hematopoietic stem cell (HSC) and (**b**) human multipotent progenitors (MPP). Vertical hash marks indicate points of censored data. Both cell types were significantly associated with patient survival (*P* < 0.05, log-rank test).

To further validate our results from using murine lineages, we examined the predictive ability of analogous human lineage profiles from both the GSE24006 dataset by Gentles *et al.*^19^ and the GSE24759 dataset by Novershtern *et al.*^34^ The Gentles *et al.* dataset included transcriptional profiles from seven different lineages: AML LSCs, AML leukemia progenitor cells, AML blasts, normal hematopoietic stem cells (HSCs), normal multipotent progenitors (MPPs), normal common myeloid progenitors, normal granulocyte-monocyte progenitors, and megakaryocyte-erythrocyte progenitors. We calculated LSSs for the seven cell types for each patient and then correlated the LSSs with survival using a univariate Cox proportional-hazards model, just as we did for the mouse data (Supplementary Table S3). Both the human HSC and MPP expression profiles were significantly correlated with patient survival (*P* = 0.001, HR = 1.04, 95% CI = 1.02–1.07, and *P* = 0.006, HR = 1.02, 95% CI = 1.01–1.04, respectively, Cox PH). Figure 7A shows the survival distributions of samples from the high and low HSC LSS groups (*P* = 0.002, log-rank test). This analysis is repeated with the MPP LSSs in Fig. 7B (P = 0.005, log-rank test).

The Novershtern *et al.* dataset detailed a comprehensive gene expression analysis of 38 different cell types involved in human hematopoiesis. To examine the analogous cell types’ association with patient survival, we repeated the analysis we performed on the Gentles *et al.* data, and examined the results in the relevant dedifferentiated cell populations (Supplementary Table S4). Three of the four dedifferentiated cell types were significantly associated with poor survival, the CD133^+^CD34 diminished HSCs (*P* = 0.03, HR = 1.04, 95% CI = 1.00–1.08, Cox PH), CD38^–^CD34^+^ HSCs (*P* = 0.02, HR = 1.03, 95% CI = 1.01–1.06, Cox PH), and megakaryocyte erythroid progenitors (*P* = 0.005, HR = 1.09, CI = 1.03–1.16, Cox PH). The fourth dedifferentiated cell type, the common myeloid progenitor, was not significantly associated with patient survival (*P* > 0.05, Cox PH), matching our findings using the murine data and the dataset by Gentles *et al.*

In both cases, these findings were in agreement with our results using murine lineage profiles, as human HSC lineages were analogous to the mouse STRSC line^23^. Additionally, the findings in both datasets globally showed a pattern consistent with our findings using the univariate Cox model (see Supplementary Table S1), with LSSs from dedifferentiated murine cell types being predictive of patient survival. The reproducibility of our findings in both mouse and humans suggested that the murine lineage profiles generated by the Immunological Genome Project were useful proxies for the analogous human immune cell lineages and had potential clinical applications in the context of human AML.

## Discussion

AML is a heterogeneous disease that presents prognostic challenges. Beyond differences in cytogenetics and specific gene mutations, it is increasingly understood that a major source of AML’s variation in survival is a result of gene expression programs inherited from the origin cell of an LSC. This suggests that a better understanding of hematopoietic transcriptional profiles in the context of AML would provide additional insight into AML patient survival outcome. This is the general method we pursued here, utilizing murine hematopoietic transcription profiles for their high quality, high resolution, and similarity to human hematopoietic profiles. Our integrative analysis compared the gene expression profiles of AML samples to the gene expression profiles of 232 murine hematopoietic cell lineages, quantifying the degree of concordance between the AML samples and cell lineages using the Lineage Similarity Score (LSS). These scores allowed us to assess the activity of hematopoietic programs in human AML at a higher resolution than previously reported, and together with Cox PH models, examine each cell type’s association with patient survival to identify which were survival prognostic. In agreement with previous literature, transcriptional similarity to immature hematopoietic cells tended to be associated with shorter survival times than transcriptional similarity to more differentiated cell types^18^,^19^,^20^,^21^.

To demonstrate the utility of our analysis, we highlighted the murine STRSC, the mouse analog of the human HSC, as a prognostic indicator. By using the LSSs of this profile as a continuous variable to predict patient survival, we achieved a hazard ratio of 1.17, which compares favorably to the LSC signature developed by Gentles *et al.* (HR = 1.15)^19^. Our profile remained predictive even after correcting for clinical and molecular pathological variables. The STRSC LSS was predictive across multiple datasets and correlated with existing AML classification schemes, as significant differences were found between FAB subtypes and *FLT3* mutation groups. Furthermore, STRSC LSSs were correlated with response to induction therapy, with a lower LSS being characteristic of a complete response. Taken together, these data showed that the LSS serves as a useful prognostic indicator that can be used to elucidate the underlying gene expression program of a patient’s disease.

Correlating the large number of murine hematopoietic gene expression profiles to AML gene expression data yielded promising results for the future of AML classification and prognosis. The resolution at which the murine hematopoietic system has been characterized to date has not yet been achieved in humans. As such, future applications that use well-characterized murine genomic data may introduce new avenues in disease characterization. The reproducible results we obtained by using murine hematopoietic cell profiles, and the similarity between our findings in murine and human profiles, demonstrate that this data is applicable to human hematologic disease and may have further applications to areas of computational genomics, and hematology and oncology in general. However, as important differences still remain between murine and human hematopoietic lineages^23^, the method presented in this paper will likely be improved further as our capacity to profile human hematopoietic cells catches up to our murine profiling ability. Additionally, as more comprehensive datasets emerge that measure the full array of cytogenetic abnormalities used to classify AML patients, a better understanding of the relationship between hematopoietic transcriptional profiles and traditional prognostic indicators can be obtained. This understanding could allow for further characterization of the underlying biology of AML, and aid in personalized therapeutic research efforts.

Going forward, a major focus will be on better understanding the role of hematopoietic transcriptional profiles across other cancers that arise from the misregulation of hematopoiesis similarly to AML, such as acute lymphoblastic leukemia (ALL). Our analysis can be readily applied to these cancers to identify prognostic hematopoietic profiles assuming there is gene expression data and survival information present for a given cohort of patients. Similarly to AML, the results of this analysis in other hematopoietic diseases can aid in disease classification and subtyping, enabling personalized therapeutic approaches. Additionally, the results obtained using this method can be easily compared across diseases, which can help distinguish important similarities and differences about the hematopoietic state of each one.

In conclusion, we have developed an integrative analysis that correlates murine hematopoietic cell gene expression profiles to AML patient data to measure the concordance of their gene expression programs. Using this technique, we have found, in agreement with other literature, that transcriptional similarity to less differentiated hematopoietic cell types is indicative of a poor prognosis. We demonstrate this using the murine short-term reconstituting stem cell (STRSC), which is an especially effective predictor of clinical outcome. This effect is pronounced in the unique LSS distribution that can be used to define traditional AML subgroups and treatment response groups. Additionally, our findings using murine profiles are reproducible in several datasets, and our methodology finds similar results when substituting murine profiles for human ones. In summary, our analysis provides a method to assess the role of hematopoietic transcriptional programs in AML patient survival. We are hopeful going forward that the results of this analysis can eventually be translated into useful clinical practice.

## Methods

### Datasets

Five AML gene expression datasets were analyzed in this study. Each dataset chosen contained at least 100 samples and included clinical data and sufficient overall survival outcome information. Three of the datasets were obtained from the Gene Expression Omnibus (GEO) database under the accession numbers GSE425 (Bullinger *et al.*, n = 116)^27^, GSE1159 (Valk *et al.*, n = 285)^15^, and GSE12417 (Metzeler *et al.*, n = 242)^32^. Acute myeloid leukemia (LAML) Level 3 gene expression data and clinical information was downloaded from The Cancer Genome Atlas data portal (n = 171)^33^. The remaining dataset was obtained from the NCI caArray database under the accession number willm-00119 (Wilson *et al.*, n = 170)^31^.

Mouse immune lineage gene expression profiles (n = 232) from the Immunological Genome Project were downloaded on 7/22/14 from the GEO database under the accession number GSE15907^24^. Human immune lineage gene expression profiles were downloaded from the GEO database under the accession number GSE24006 (Gentles *et al.*, n = 8)^19^ and GSE24759 (Novershtern *et al.*, n = 38)^34^. For the willm-00119 and GSE15907 datasets (.CEL files), the data was background corrected using Robust Microarray Analysis (RMA), quantile normalized, and fitted with a multichip linear model for each probeset. These techniques were carried out using the “expresso” function of the “affy” library in R^35^. GSE12417, which contained gene expression measurements from the GPL96, GPL97, and GPL570 platforms, was represented by the probeset overlap between GPL96 and GPL570. For all datasets, probeset expression was converted into gene expression. Genes with multiple probesets were represented by the probeset with the highest average intensity across all samples. Murine transcripts were matched to human transcripts on the basis of gene symbol.

### Pre-processing of hematopoietic cell profiles

To calculate the LSS, the BASE algorithm^25^ requires that hematopoietic cell expression profiles be normalized to reflect the relative expression of each gene in a given cell type. This process can be broken into five steps: **(i)** Gene expression values in the dataset containing murine or human hematopoietic profiles (GSE15907, GSE24006, or GSE24759) are median normalized across cell types to transform absolute expression values into relative expression values. As a result, genes that are more highly expressed in a given cell type relative to the other cell types will have a higher expression value. **(ii)** Each cell type’s median-normalized values are then z-transformed, causing each cell type’s expression values to follow a standard normal distribution (~N(0,1)). Z-scores > 0 indicate genes that are up-regulated in a given cell type relative to the other cell types in the dataset, while z-scores < 0 indicate the opposite. In some cases, datasets may contain replicate gene expression experiments representing the same cell type that need to be combined into a single column. These replicates are collapsed during this step, using the mean z-score of each replicate and then each cell type’s values are z-transformed again to renormalize. **(iii)** Each cell type’s resulting z-scores are then transformed into up- and down-regulated subsets. In the up-regulated subset, z-scores > 0 maintain their value, while z-scores < 0 are converted to 0, while the opposite is done in the down-regulated subset. This process allows for the transformation of z-scores into p-values without losing the relative expression information contained in the z-scores. **(iv)** The p-values are then –log10 transformed to place greater weight on differentially expressed genes. Outliers defined as transformed values > 10 are then trimmed to a maximum of 10. **(v)** The resulting values are then scaled to values between 0 and 1 by dividing each value by the maximum value in the overall dataset.

### Calculation of the LSS

To calculate the LSS, the BASE algorithm is inputted with the transformed hematopoietic cell profiles and patient gene expression data. The up- and down-regulated subsets of each hematopoietic cell type’s transformed values are defined as a weight vector **W** = [*w*_1_, *w*_2_, *w*_3_…*w*_*j*_…*w*_*n*_]; where *w*_*j*_ = −log_10_(p-val) for gene *j* in that cell lineage and *n *= # of genes. Patient gene expression data is then processed based on the microarray platform. Gene expression values from two-channel arrays require no additional processing, but one-channel arrays must first be log-transformed and median normalized across samples, so that each gene expression value will reflect relative expression between patients. Each patient’s gene expression profile is then treated as the vector **g** = [*g*_1_, *g*_2_, *g*_3_…*g*_*j*_…*g*_*n*_], which contains sorted (decreasing) gene expression values for each gene *g*_*j*_. Using these vectors, the BASE algorithm calculates a “pre-LSS” for the “up” (pLSS_up_) and “down” (pLSS_dn_) lineage subsets through the following steps:

First, a foreground function (f(i)) is used to calculate the cumulative distribution of the gene expression values for each patient weighted by their corresponding transformed hematopoietic relative expression values:




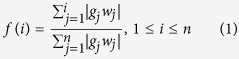




Second, a background function (b(i)) is used to calculate the cumulative distribution of the gene expression values for each patient weighted by a value complementary to the transformed hematopoietic relative expression values:




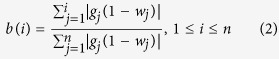




The maximum deviation of these two functions can then be compared to determine the pLSS_up_ and pLSS_dn_. This deviation is calculated by subtracting the foreground function from the background function and taking the difference with the largest absolute value. This requires comparing the maximum difference when the foreground function is larger than the background function (pLSS^+^) to the minimum difference when the background function is larger than the foreground function (pLSS^−^). This process is performed for both the up- and down-regulated subsets of each hematopoietic cell type. This step is formulized below:




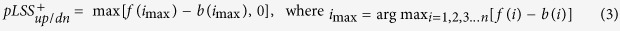







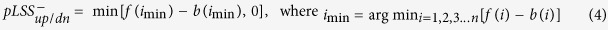







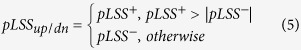




In the case of the up-regulated hematopoietic subset, a hematopoietic cell type that is highly similar to a patient gene expression profile will cause the foreground function to increase quickly in the beginning, as highly expressed patient genes are assigned high hematopoietic weights, before plateauing towards the end, as lowly expressed patient genes are assigned low weights. The background function will increase slowly at the beginning, as lowly expressed genes are assigned low complementary weights, before increasing quickly at the end. In the case of the down-regulated hematopoietic subset, where higher weights correspond to more lowly expressed hematopoietic genes, the foreground function will behave like the background function did in the up-regulated set and vice versa. As a result, in the case of a highly similar patient profile and hematopoietic cell type, there will be a high deviation between the foreground and background functions for both the up-regulated and down-regulated subsets, resulting in a high pLSS_up_ and pLSS_dn_. In the case of low similarity between the patient gene expression profile and hematopoietic profile, both the foreground and background functions will be expected to increase randomly, which would result in a low maximum deviation between the two functions, and thus a low pLSS_up_ and pLSS_dn_.

After the pLSS_up_ and pLSS_dn_ are calculated they are then normalized to their respective null distributions. The null distribution is generated by permuting the gene labels in the patient ranked gene list 1000 times and recalculating the pLSS_up_ or pLSS_dn_ for each permutation using equations 1–5. The pLSS_up_ and pLSS_dn_ are then divided by the mean of the absolute value of the permuted values to yield the LSS_up_ and LSS_dn_. For datasets derived from two-channel arrays, the final LSS was obtained by subtracting LSS_dn_ from LSS_up_. For one-channel array datasets, which measure absolute mRNA expression, the LSS_up_ served as the final LSS, as the LSS_dn_ was enriched in genes whose low expression values were obscured by noise from nonspecific cross hybridization.

### Survival analyses

Univariate Cox PH models were fitted to the LSSs for each lineage across all samples in a dataset to investigate the relationship between LSS and survival time. For survival-associated LSSs, multivariate Cox PH models incorporating FAB subtype, *FLT3* mutation status, age, CALGB cytogenetic risk^36^, and history of preceding malignancy were additionally constructed. Significance of the model parameters was assessed using the Wald test and p-values were adjusted using the Benjamini-Hochberg procedure. Kaplan-Meier curves were used to visualize the results, with samples stratified into two groups based on their LSS scores: LSSs that were distributed bimodally were dichotomized about the local minima between the two peaks, otherwise, samples were dichotomized about their modal frequency. A log-rank test was used to estimate the significance of the difference between the survival curves.

Survival analyses were performed in R using the “survival” package’s “coxph”, “survfit”, and “survdiff” functions for Cox PH modeling, Kaplan-Meier plotting, and log-rank significance testing, respectively.

## Additional Information

**How to cite this article**: Varn, F. S. *et al.* Systematic analysis of hematopoietic gene expression profiles for prognostic prediction in acute myeloid leukemia. *Sci. Rep.* **5**, 16987; doi: 10.1038/srep16987 (2015).

## Supplementary Material

Supplementary Information
