# Supplementary material for: Systematic analysis of hematopoietic gene expression profiles for prognostic prediction in acute myeloid leukemia
Source: Sci Rep. 2015 Nov 24;5:16987. doi: 10.1038/srep16987 (PMC4657053; doi:10.1038/srep16987)
Supplement: Supplementary Information [file srep16987-s1.pdf]

Supplementary Information

Systematic analysis of hematopoietic gene expression profiles for prognostic prediction in acute myeloid leukemia

Frederick S. Varn, Erik H. Andrews, Chao Cheng

Supplementary Figure S1: Page 2

Supplementary Table S1: Page 3

Supplementary Table S2: Page 9

Supplementary Table S3: Page 10

Supplementary Table S4: Page 11

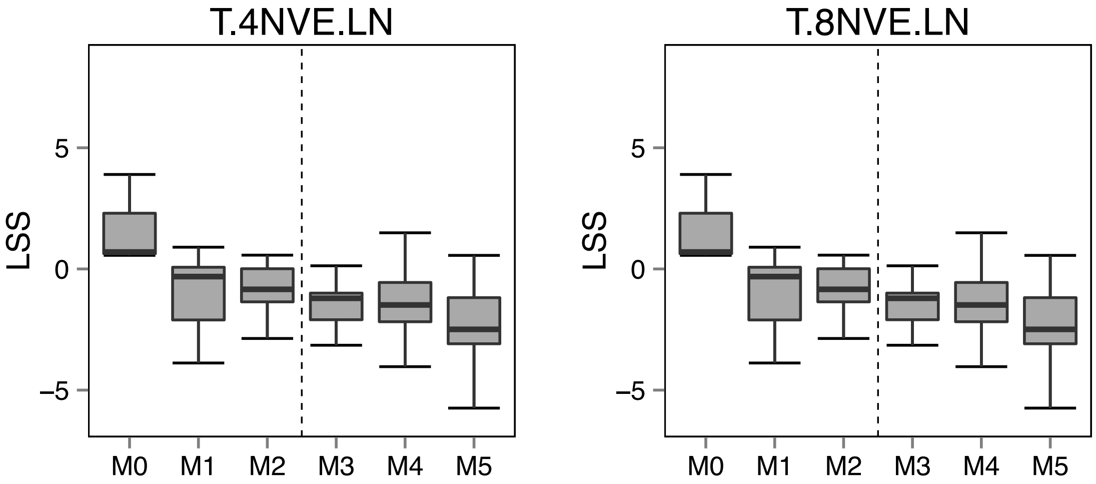

Supplementary Figure S1. Association of naïve T Cell LSS with FAB subtype. Distributions of the CD4+ naïve T cell (left) and CD8+ naïve T cell (right) LSS across the different FAB subtypes (M0-M5) available in the Bullinger dataset. There was a significant difference in LSS between the subtypes for both cell types (*P* = 4e-3 and 4e-4, Kruskal-Wallis, CD4+ naïve T cell and CD8+ naïve T cell, respectively). For all panels, box spans quartiles, with line representing median. Outliers were not removed. Whiskers represent absolute range excluding outliers.

Supplementary Table S1. Univariate Cox PH Models for Hematopoietic Cell Types

Name	P-val	Adj.p-val	HR	95% CI
CD150negCD48neg.BM	4.17E-05	0.01	1.17	1.08 - 1.25
NKT.44posNK1.1pos.TH	2.84E-04	0.03	0.81	0.72 - 0.91
TGD.VG5pos.IEL.	8.92E-04	0.07	0.73	0.60 - 0.88
LTHSC.FL	2.08E-03	0.08	1.09	1.03 - 1.14
T.DN1neg2.TH	2.39E-03	0.08	1.27	1.09 - 1.48
X0negSTnegV1_SC.LT34F.BM	2.45E-03	0.08	1.06	1.02 - 1.10
T.ETP.TH	2.72E-03	0.08	1.21	1.07 - 1.36
MLP.BM	2.76E-03	0.08	1.08	1.03 - 1.14
CD150negCD48neg.FL	2.84E-03	0.08	1.09	1.03 - 1.16
TGD.VG5pos.ACT.IEL.	0.01	0.13	0.81	0.70 - 0.94
X0negSTnegV1_DC.103neg11Bpos.LULN	0.01	0.19	0.92	0.86 - 0.98
X0negSTnegV1_DC.PDC.8pos.SLN	0.01	0.21	0.79	0.66 - 0.95
DC.LC.SK	0.01	0.21	0.96	0.93 - 0.99
DC.8neg4neg11Bpos.SLN	0.01	0.21	0.88	0.79 - 0.97
X0negSTnegV1_DC.103pos11Bneg.LULN	0.01	0.21	0.96	0.93 - 0.99
T.8EFF.SP.OT1.D8.VSVOVA	0.02	0.23	0.92	0.85 - 0.98
NK.Hpos.MCMV1.SP	0.02	0.23	0.77	0.61 - 0.95
DC8.LN	0.02	0.23	0.90	0.83 - 0.98
LTHSC.BM	0.02	0.23	1.15	1.02 - 1.29
MO.6CposIIneg.LN	0.02	0.24	0.96	0.93 - 0.99
DC7.LN	0.02	0.24	0.91	0.85 - 0.99
X0negSTnegV1_DC.PDC.8pos.MLN	0.02	0.24	0.82	0.70 - 0.97
NK.Hpos.MCMV7.SP	0.02	0.24	0.87	0.78 - 0.98
DC.8neg4neg11Bneg.SLN	0.02	0.24	0.93	0.88 - 0.99
X0negSTnegV1_B6.14W.ABD.TR	0.03	0.25	0.89	0.80 - 0.99
CLP	0.03	0.25	1.10	1.01 - 1.21
X0negSTnegV1_SC.ST34F.BM	0.03	0.28	1.06	1.01 - 1.11
DC4.MLN	0.03	0.29	0.90	0.82 - 0.99
DC.103pos.11Bneg.POLYIC.LU	0.04	0.29	0.95	0.90 - 1.00
TGD.VG5neg.IEL	0.04	0.29	0.89	0.79 - 0.99
NK.MCMV7	0.04	0.30	0.88	0.78 - 0.99
DC9.LN	0.04	0.30	0.96	0.92 - 1.00
X0negSTnegV1_DC.11CLOSER.SALM3.SI	0.04	0.30	0.94	0.89 - 1.00
X0negSTnegV1_DC.103neg11Bpos.SALM3.SI	0.04	0.30	0.93	0.87 - 1.00
DC.103CLOSER.SI	0.05	0.31	0.89	0.79 - 1.00
DC3.MLN	0.05	0.31	0.94	0.89 - 1.00
PROB.FRA.FL	0.05	0.31	1.06	1.00 - 1.12
NKT.4pos.SP	0.05	0.31	0.94	0.89 - 1.00
DC.103neg.11Bpos.POLYIC.LU	0.05	0.31	0.95	0.91 - 1.00
DC.8neg4neg11Bpos.MLN	0.05	0.31	0.94	0.87 - 1.00

X0negSTnegV1_GN.ARTH.SYNF	0.05	0.31	0.96	0.93 - 1.00
PREB.FRD.FL	0.06	0.31	1.07	1.00 - 1.14
T.DN2.TH	0.06	0.31	1.06	1.00 - 1.12
X0negSTnegV1_SC.MPP34F.BM	0.06	0.31	1.07	1.00 - 1.14
X0negSTnegV1_T.DN1neg2.TH	0.06	0.33	1.06	1.00 - 1.12
DC.103neg11Bpos.LV	0.06	0.34	0.97	0.94 - 1.00
X0negSTnegV1_SC.MEP.BM	0.07	0.36	1.05	1.00 - 1.11
NK.HposMCMV1	0.07	0.36	0.86	0.74 - 1.01
X0negSTnegV1_DC.103pos11Bpos.SALM3.SI	0.07	0.36	0.95	0.89 - 1.01
T.8EFF.SP.OT1.D45VSV	0.07	0.36	0.90	0.80 - 1.01
DC.8neg4neg11Bneg.MLN	0.08	0.36	0.96	0.92 - 1.00
MLP.FL	0.08	0.36	1.06	0.99 - 1.13
TGD.VG2neg.ACT.SP	0.08	0.36	0.97	0.93 - 1.00
TGD.SP	0.08	0.36	0.97	0.94 - 1.00
PROB.CLP.FL	0.08	0.36	1.05	0.99 - 1.11
B614WABDTREG	0.09	0.36	0.95	0.90 - 1.01
DC.103pos11Bpos.SI	0.09	0.36	0.91	0.81 - 1.01
DC.103neg11Bpos.SI	0.09	0.36	0.90	0.80 - 1.02
DC2.LU	0.10	0.38	0.98	0.95 - 1.00
T.4MEM44H62L.SP	0.10	0.38	0.89	0.78 - 1.02
TGD.VG2neg.SP.TCRBKO	0.10	0.38	0.96	0.90 - 1.01
T.8EFF.SP.OT1.D10.LISOVA	0.10	0.38	0.93	0.84 - 1.02
X0negSTnegV1_MF.MEDL.SLN	0.11	0.38	0.99	0.99 - 1.00
X0negSTnegV1_DC.103neg11Bpos24pos.LU	0.11	0.38	0.99	0.97 - 1.00
DC1.SP	0.11	0.38	0.91	0.82 - 1.02
NK.SP	0.11	0.38	0.94	0.88 - 1.01
MO.6CnegIIpos.BL	0.11	0.38	0.97	0.94 - 1.01
X0negSTnegV1_GN.BL	0.11	0.38	0.96	0.91 - 1.01
X0negSTnegV1_MF.169pos11CHI.SLN	0.11	0.38	0.98	0.96 - 1.00
NKT.4neg.LV	0.11	0.38	0.94	0.88 - 1.01
MICROGLIA.CNS	0.12	0.38	0.97	0.94 - 1.01
MO.6CnegIIneg.BM	0.12	0.38	0.98	0.96 - 1.00
NKT.4neg.SP	0.12	0.38	0.94	0.86 - 1.02
X0negSTnegV1_MF.SBCAPS.SLN	0.12	0.38	0.99	0.97 - 1.00
DC.11CLOSER.SI	0.12	0.38	0.92	0.82 - 1.02
DC.8neg4neg11Bneg.SP	0.12	0.39	0.92	0.83 - 1.02
X0negSTnegV1_GN.ARTH.BM	0.12	0.39	0.95	0.90 - 1.01
DC.PDC.8neg.SP	0.13	0.40	0.95	0.88 - 1.02
X0negSTnegV1_GN.THIO.PC	0.13	0.40	0.96	0.91 - 1.01
X0negSTnegV1_GN.URAC.PC	0.13	0.40	0.96	0.90 - 1.01
DC2.SP	0.14	0.40	0.93	0.84 - 1.02
PROB.FRBC.FL	0.14	0.40	1.05	0.99 - 1.11
MO.6CposIIpos.BL	0.14	0.40	0.95	0.89 - 1.02

PROB.FRBC.BM	0.14	0.40	1.05	0.98 - 1.13
T.8EFF.SP.OT1.D8.LISOVA	0.14	0.40	0.95	0.88 - 1.02
TGD.VG2pos.SP.TCRBKO	0.14	0.40	0.97	0.93 - 1.01
NK.49CIpos.SP	0.15	0.41	0.96	0.90 - 1.02
PROB.CLP.BM	0.15	0.41	1.05	0.98 - 1.13
T.8NVE.SP	0.15	0.41	0.96	0.92 - 1.01
IMMTGD.VG2pos.TH	0.15	0.41	1.07	0.98 - 1.16
X0negSTnegV1_DC.103pos11Bneg.LU	0.16	0.41	0.94	0.87 - 1.02
DC.8neg.TH	0.16	0.42	0.96	0.91 - 1.02
MO.6CnegIIINT.BL	0.16	0.42	0.96	0.90 - 1.02
T.DN3B.TH	0.17	0.44	1.06	0.98 - 1.14
MO.6CposIIneg.BL	0.17	0.44	0.95	0.88 - 1.02
T.8EFF.SP.OT1.D15LIS	0.19	0.46	0.94	0.87 - 1.03
X0negSTnegV1_T.DN2B.TH	0.19	0.46	1.04	0.98 - 1.10
X0negSTnegV1_SC.CMP.BM.DR	0.19	0.46	1.04	0.98 - 1.11
MO.6CnegIIneg.BL	0.19	0.46	0.97	0.93 - 1.01
MO.6CposIIneg.BM	0.20	0.46	0.98	0.96 - 1.01
T.DN3neg4.TH	0.20	0.46	1.05	0.97 - 1.14
MATTGD.VG1pos.TH.B6	0.20	0.46	0.96	0.91 - 1.02
X0negSTnegV1_DC.103neg11Bpos24neg.LU	0.20	0.46	0.99	0.99 - 1.00
DC6.LN	0.20	0.46	0.98	0.95 - 1.01
NK.49CIneg.SP	0.20	0.46	0.96	0.90 - 1.02
DC.103pos11Bneg.SI	0.20	0.46	0.95	0.88 - 1.03
DC2.MLN	0.21	0.46	0.98	0.95 - 1.01
DC1.LN	0.21	0.46	0.96	0.90 - 1.02
DC.103pos11Bneg.LV	0.21	0.46	0.98	0.95 - 1.01
B.T2.SP	0.21	0.46	1.05	0.97 - 1.14
X0negSTnegV1_T.DN2A.TH	0.22	0.46	1.02	0.99 - 1.05
T.DN2neg3.TH	0.22	0.46	1.04	0.98 - 1.11
T.8EFF.SP.OT1.D8LISO	0.22	0.46	0.91	0.78 - 1.06
T.8EFF.SP.OT1.D15.LISOVA	0.23	0.46	0.97	0.93 - 1.02
NK.DAP12neg.SP	0.23	0.46	0.92	0.80 - 1.05
GN.BM	0.23	0.46	0.95	0.88 - 1.03
T.4MEM.SP	0.23	0.46	0.93	0.82 - 1.05
DC3.SP	0.23	0.46	0.98	0.95 - 1.01
FRC.MLN	0.23	0.46	0.95	0.88 - 1.03
X0negSTnegV1_T.ETP.TH	0.24	0.47	1.02	0.99 - 1.05
TGD.TH	0.24	0.47	1.06	0.96 - 1.18
MF.THIO5.IIpos480INT.PC	0.24	0.47	0.98	0.94 - 1.02
FRC.SLN	0.25	0.48	0.95	0.88 - 1.03
NK.49Hneg.SP	0.25	0.48	0.93	0.82 - 1.05
MF.LU	0.25	0.48	0.97	0.93 - 1.02
T.ISP.TH	0.25	0.48	1.04	0.97 - 1.12

T.8MEM.SP.OT1.D45.LISOVA	0.26	0.48	0.93	0.82 - 1.05
MF.RP.SP	0.26	0.48	0.93	0.82 - 1.06
T.DN3A.TH	0.27	0.49	1.05	0.96 - 1.14
EP.MECHI.TH	0.27	0.50	0.98	0.95 - 1.02
FI.SK	0.27	0.50	0.97	0.91 - 1.03
IMMTGD.VG2.E17.TH	0.28	0.50	1.05	0.96 - 1.14
T.8MEM.SP.OT1.D106.VSVOVA	0.28	0.50	0.98	0.94 - 1.02
T.8EFF.SP.OT1.D6.VSVOVA	0.28	0.50	0.96	0.90 - 1.03
T.DPBL.TH	0.29	0.51	1.04	0.97 - 1.11
NK.DAP10neg.SP	0.29	0.51	0.94	0.85 - 1.05
X0negSTnegV1_B6.14W.LN.TR	0.29	0.51	0.91	0.76 - 1.09
X0negSTnegV1_B.FO.LN	0.30	0.51	0.89	0.72 - 1.11
DC.PDC.8pos.SP	0.30	0.51	0.97	0.90 - 1.03
T.8EFF.SP.OT1.D15.VSVOVA	0.31	0.53	0.96	0.89 - 1.04
DC2.LN	0.32	0.53	0.99	0.97 - 1.01
X0negSTnegV1_T.8EFF.SP.OT1.24HR.LISOVA	0.32	0.53	1.03	0.97 - 1.10
TGD.VG2pos.ACT.SP	0.33	0.55	0.98	0.95 - 1.02
X0negSTnegV1_ST.31neg38neg44neg.SLN	0.34	0.56	0.98	0.93 - 1.03
X0negSTnegV1_DC.103pos11Bneg.SALM3.SI	0.35	0.57	0.95	0.85 - 1.06
B.FRE.BM	0.35	0.57	0.89	0.69 - 1.14
MF.THIO5.IIneg480HI.PC	0.36	0.58	0.98	0.95 - 1.02
IMMTGD.VG1posVD6pos.TH.B6	0.37	0.59	1.04	0.95 - 1.14
IMMTGD.VG1pos.TH.B6	0.38	0.60	1.04	0.95 - 1.14
MF.THIO5.IIneg480INT.PC	0.38	0.60	0.98	0.93 - 1.03
MATTGD.VG3.E17.TH	0.39	0.61	1.03	0.96 - 1.11
X0negSTnegV1_MATTGD.VG2pos.TH	0.39	0.61	1.03	0.96 - 1.10
PREB.FRC.BM	0.40	0.61	1.03	0.96 - 1.11
X0negSTnegV1_TGD.VG5pos24AHI.TH	0.40	0.61	1.03	0.96 - 1.11
DC1.MLN	0.40	0.61	0.97	0.91 - 1.04
FI.MTS15pos.TH	0.40	0.61	0.97	0.89 - 1.05
T.4.LN.BDC	0.41	0.61	0.95	0.84 - 1.07
TGD.VG5pos.ACT.IEL	0.43	0.64	0.96	0.86 - 1.07
DC.8pos.TH	0.43	0.64	0.98	0.93 - 1.03
T.4NVE.MLN	0.44	0.64	0.97	0.91 - 1.04
TGD.VG5neg.ACT.IEL	0.44	0.64	0.91	0.72 - 1.15
T.8MEM.LN	0.45	0.65	0.98	0.92 - 1.04
T.DN4.TH	0.45	0.65	1.03	0.96 - 1.10
B.T3.SP	0.45	0.65	1.03	0.96 - 1.10
B6SPLFO	0.45	0.65	1.03	0.96 - 1.11
T.8EFF.SP.OT1.D6LISO	0.46	0.65	1.01	0.98 - 1.05
X0negSTnegV1_DC.103neg11BposF4neg80LO.KD	0.46	0.65	0.98	0.91 - 1.04
T.4NVE.LN	0.46	0.65	0.96	0.85 - 1.08
T.4.PLN.BDC	0.48	0.67	0.96	0.86 - 1.08

BEC.MLN	0.50	0.69	0.95	0.84 - 1.09
X0negSTnegV1_SC.CDP.BM	0.52	0.71	0.98	0.94 - 1.03
X0negSTnegV1_T.8EFF.SP.OT1.48HR.LISOVA	0.52	0.71	1.02	0.97 - 1.07
PROB.FRA.BM	0.52	0.71	1.02	0.96 - 1.08
T.8MEM.SP.OT1.D100.LISOVA	0.52	0.71	0.95	0.83 - 1.10
T.4FP3pos25pos.SP	0.53	0.71	0.98	0.91 - 1.05
BEC.SLN	0.53	0.71	0.96	0.84 - 1.09
B1A.PC	0.53	0.71	0.92	0.70 - 1.20
X0negSTnegV1_TGD.VG3pos24AHI.E17.TH	0.55	0.72	1.02	0.95 - 1.11
X0negSTnegV1_T.8EFF.SP.OT1.12HR.LISOVA	0.56	0.73	1.03	0.94 - 1.12
T.4FP3neg.SP	0.58	0.75	0.97	0.89 - 1.07
PREB.FRD.BM	0.58	0.75	1.02	0.95 - 1.09
B.GC.SP	0.59	0.76	1.02	0.94 - 1.11
MF.THIO5.IIpos480LO.PC	0.62	0.79	0.99	0.94 - 1.04
FRA	0.62	0.79	1.01	0.96 - 1.06
B614WLNTREG	0.63	0.80	0.97	0.85 - 1.11
T.8MEM.SP	0.66	0.83	1.03	0.91 - 1.16
NK.B2Mneg.SP	0.66	0.83	0.98	0.87 - 1.09
T.8NVE.PP	0.67	0.84	0.99	0.94 - 1.04
MF.IIneg480HI.PC	0.68	0.84	0.99	0.95 - 1.04
X0negSTnegV1_SC.MDP.BM	0.69	0.85	1.01	0.96 - 1.06
NKT.44negNK1.1neg.TH	0.70	0.85	1.02	0.92 - 1.12
LEC.SLN	0.70	0.85	0.97	0.84 - 1.13
B.T1.SP	0.71	0.85	0.95	0.74 - 1.23
X0negSTnegV1_B.FO.MLN	0.72	0.86	0.95	0.71 - 1.27
T.4MEM44H62L.LN	0.72	0.86	0.98	0.88 - 1.09
T.8EFF.SP.OT1.D5.VSVOVA	0.72	0.86	0.99	0.93 - 1.05
T.8NVE.LN	0.72	0.86	0.98	0.89 - 1.08
MF.BM	0.74	0.86	0.99	0.96 - 1.03
TGD.VG2pos.SP	0.74	0.86	1.01	0.94 - 1.09
B1A.SP	0.74	0.86	0.97	0.80 - 1.17
B.FRE.FL	0.75	0.87	0.97	0.83 - 1.14
LEC.MLN	0.76	0.88	0.98	0.84 - 1.14
NK.49Hpos.SP	0.79	0.91	0.98	0.88 - 1.11
T.8EFF.SP.OT1.D10LIS	0.80	0.92	1.02	0.87 - 1.20
T.4.PA.BDC	0.82	0.92	1.01	0.92 - 1.10
T.8SP69pos.TH	0.82	0.92	0.99	0.92 - 1.07
MATTGD.VG1posVD6pos.TH.B6	0.84	0.94	0.99	0.89 - 1.09
T.4INT8pos.TH	0.84	0.94	1.01	0.93 - 1.09
NKT.4pos.LV	0.85	0.94	1.02	0.85 - 1.22
B.FO.PC	0.85	0.94	0.99	0.89 - 1.10
B.FRF.BM	0.86	0.94	0.99	0.85 - 1.15
T.4NVE.SP	0.86	0.94	1.01	0.95 - 1.07

T.4MEM.LN	0.86	0.94	0.99	0.92 - 1.07
T.8SP24neg.TH	0.86	0.94	1.01	0.92 - 1.10
T.8SP24INT.TH	0.87	0.94	1.01	0.92 - 1.11
T.4NVE.PP	0.88	0.94	1.01	0.92 - 1.10
T.8EFF.SP.OT1.D6.LISOVA	0.88	0.94	0.99	0.93 - 1.07
T.8NVE.MLN	0.89	0.94	0.99	0.88 - 1.11
T.4pos8INT.TH	0.90	0.95	1.00	0.93 - 1.08
NKT.44posNK1.1neg.TH	0.93	0.98	1.00	0.91 - 1.09
T.DPSM.TH	0.93	0.98	1.00	0.89 - 1.11
TGD.VG2neg.SP	0.94	0.98	1.00	0.93 - 1.09
B1B.PC	0.96	0.99	1.00	0.92 - 1.08
DC.IIpos480LO.PC	0.96	0.99	1.00	0.95 - 1.05
T.DP.TH	0.97	0.99	1.00	0.92 - 1.08
GMP.BM	0.97	0.99	1.00	0.96 - 1.04
B.MZ.SP	0.98	1.00	1.00	0.77 - 1.28
T.4SP24INT.TH	0.99	1.00	1.00	0.91 - 1.10
T.DP69pos.TH	0.99	1.00	1.00	0.92 - 1.09
T.4SP24neg.TH	0.99	1.00	1.00	0.92 - 1.09
T.4SP69pos.TH	1.00	1.00	1.00	0.91 - 1.09
T.8NVE.SP.OT1	1.00	1.00	1.00	0.95 - 1.05

Results from a univariate Cox PH model correlating LSSs from each murine hematopoietic cell type with patient survival. Cell types are arranged in ascending order of p-value.

Supplementary Table S2: STRSC Multivariate Cox PH Model

Covariate	HR	95% CI	Z	P-val
STRSC LSS	1.12	1.01 - 1.23	2.20	0.03
FLT3 mutation	2.09	1.05 - 4.13	2.11	0.04
Age	1.02	0.99 - 1.04	1.48	0.14
FAB subtype	0.83	0.67 - 1.04	-1.59	0.11
Cytogenetic risk, intermediate: favorable	1.49	0.71 - 3.13	1.05	0.29
Cytogenetic risk, poor: favorable	2.94	1.09 - 7.88	2.14	0.03
Preceding malignancy	3.43	1.39 - 8.48	2.67	8.00E-03

The STRSC LSS remains predictive in a Cox PH model even after adjusting for common clinical variables including *FLT3* mutation status, age, FAB subtype, cytogenetic risk category, and preceding malignancy.

Supplementary Table S3: Univariate Cox PH Model for Human Cell Types (GSE24006)

Name	P-val	Adj p-val	HR	95% CI
HSC	1.00E-03	0.01	1.04	1.02 - 1.07
MPP	6.00E-03	0.03	1.02	1.01 - 1.04
BM.GMP	0.06	0.15	0.98	0.97 - 1.00
BM.CMP	0.14	0.29	0.99	0.98 - 1.00
MEP	0.25	0.40	0.99	0.98 - 1.01
AML.BLAST	0.65	0.78	0.99	0.96 - 1.03
AML.LSC	0.68	0.78	0.99	0.94 - 1.04
AML.LPC	0.79	0.79	0.99	0.92 - 1.07

A univariate Cox PH model reveals that the hematopoietic stem cell (HSC) and normal multipotent progenitor cell (MPP) LSSs are significantly associated with patient survival. Cell types are arranged in ascending order of p-value.

Supplementary Table S4: Univariate Cox PH Model for Dedifferentiated Human Cell Types (GSE24759)

Name	P-val	Adj p-val	HR	95% CI
Megakaryocyte.erythroid.progenitor	5.35E-03	0.02	1.09	1.03 - 1.16
Hematopoietic.stem.cell_CD38n.CD34p	0.02	0.03	1.03	1.01 - 1.06
Hematopoietic.stem.cell_CD133p.CD34dim	0.03	0.04	1.04	1.00 - 1.08
Common.myeloid.progenitor	0.59	0.59	1.00	0.99 - 1.02

A univariate Cox PH model reveals that LSSs from three out of dedifferentiated human cell types, the megakaryocyte erythroid progenitor, the CD133+ CD34 diminished HSC, and the CD38- CD34+ HSC, are significantly associated with patient survival. Cell types are arranged in ascending order of p-value.
